# Characteristics of children admitted to intensive care with acute bronchiolitis

**DOI:** 10.1007/s00431-018-3138-6

**Published:** 2018-04-13

**Authors:** Marwa Ghazaly, Simon Nadel

**Affiliations:** 1 Paediatric Intensive Care Unit, St. Mary’s Hospital, Imperial College Healthcare NHS Trust, Praed Street, London, W21NY UK; 2 Pediatric Department, Assuit University Hospital, Assuit University, Assiut, Egypt; 3 Imperial College London, London, UK

**Keywords:** Bronchiolitis, Children, Outcome, Intensive care, Ventilation, Co-morbidity, RSV

## Abstract

To assess factors associated with outcome in children admitted to paediatric intensive care (PIC) with bronchiolitis. A retrospective study of children admitted to the PICU at St Mary’s Hospital, London with bronchiolitis over a 6-year period (2011–2016). All bronchiolitis admissions < 2 years were included. Data collected particularly noted risk factors for severity, demographics, microbiology and outcome. We compared respiratory syncytial virus (RSV) with non-RSV status. Multivariate analysis was performed. Two hundred seventy-four patients were identified. Median age was 60 days (IQR 28–150 days), 63% were male, 90% were invasively ventilated and 42% were previously healthy. Pre-existing co-morbidities were present in 38%. The most frequently isolated pathogens were RSV (60%) and rhinovirus (26%). Co-infection was present in 45%, most commonly with RSV, rhinovirus and bacterial pathogens. Median length of stay (LOS) was 6 days (IQR 4.75–10). Younger age, prematurity, RSV, co-infection and co-morbidity were identified as significant risk factors for prolonged LOS. Six children died. Five of these had documented co-morbidities.

*Conclusion*: RSV causes more severe bronchiolitis than other viruses. Nearly half of children admitted to PICU with RSV were previously healthy. Current guidelines for immunoprophylaxis of RSV bronchiolitis should be re-considered.

**Table:** 

**What is Known:** *• Bronchiolitis is one of the most common reasons for unplanned PICU admission. The most common virus causing bronchiolitis is RSV* *• Bronchiolitis severe enough to require admission to PICU is associated with frequent morbidity but has low mortality.*	
**What is New:** *• RSV causes more severe bronchiolitis than other viruses.* *• Nearly half of all children admitted to PICU with RSV were previously healthy.*

## Introduction

Acute bronchiolitis is a leading cause of hospitalization in young children. Although children with bronchiolitis do not usually require hospitalization, approximately 3% of affected children are admitted to hospital [7, 30].In the developed world, infants aged < 1 year with bronchiolitis account for 18% of all paediatric hospital admissions [13]. In December 2011 in England, there were 30,451 hospital admissions for bronchiolitis [6]. Bronchiolitis admission rates in infants under 1 year have previously been estimated to be 24.2 to 31.2 per 1000 in the UK and USA, respectively [4, 12, 21].

Most children hospitalized due to acute bronchiolitis have an uneventful course [3, 15]; however, approximately 2–6% requires admission to a paediatric intensive care unit (PICU), with 2–3% of hospitalizations requiring invasive mechanical ventilation [13]. Acute bronchiolitis accounts for around 13% of PICU admissions in the UK [9], thus being a significant burden on PICU beds, with a “winter surge” in activity occurring predictably each November to February [7, 11, 12, 29].

Prior studies have identified risk factors associated with hospitalization for bronchiolitis, including prematurity, younger age, environmental factors (e.g. passive smoking, crowded household) and presence of co-morbidities (e.g. chronic pulmonary disease, congenital heart disease, immunodeficiency, neurologic disease) [4, 18, 28, 31]. However, few studies have yet described factors associated with PICU admission and outcome.

Despite the high hospitalization rate associated with bronchiolitis, it is however uncommon for bronchiolitis to cause death. Data from children with bronchiolitis due to the most commonly associated cause—respiratory syncytial virus (RSV)—notes that deaths are rare in the developed world and range from 2.9 (UK) to 5.3 (USA) per 100,000 children below 12 months [7, 30]. In October 2009 in England, there were 72 recorded deaths of children within 90 days of hospital admission for acute bronchiolitis [6].

## Aim

We aimed to investigate factors associated with outcome in children with a diagnosis of bronchiolitis admitted to a single PICU over a 6-year period, between January 2011 and December 2016.

## Methods

### Study design

We performed a retrospective observational study in children admitted to the PICU at St Mary’s Hospital, London, UK. The PICU at St Mary’s is a general PICU, with approximately 330 admissions per year.

### Inclusion criteria

Patients were identified using the electronic database of PICU admissions (Careview, Phillips, UK). We included all patients aged <2 years with a recorded diagnosis of bronchiolitis using the SNOMED diagnostic code.

### Exclusion criteria

Patients with missing records (two patients).

### Data collection

We reviewed all identified children’s medical records to collect clinical data, including from the pre-hospitalization illness, the initial presentation to the emergency department and the child’s inpatient course prior to PICU admission, including vital signs, medical management and PICU outcome. We particularly noted recognized risk factors for severity, admission data, demographic data, microbiology and outcome.

### Pathology

All children had standardized sample collection on admission to PICU. This included nasopharyngeal aspirate (NPA) for virus isolation, blood cultures, endotracheal tube (ETT) aspirate in intubated patients for bacteria and other relevant cultures depending on clinical presentation. All samples were collected and analysed using a standardized protocol: The NPA samples taken on PICU admission were tested for viruses using rapid direct immunofluorescence and/or real-time multiplex PCR for the following respiratory panel: RSV; rhinovirus; adenovirus; parainfluenza viruses 1, 2 and 3; and influenza virus A and B. Extended analyses were conducted on NPA or ETT specimens depending on advice from the Infectious Diseases Service. The results of all other bacterial cultures on respiratory specimens, urine, blood, mucous membrane cultures are also recorded.

Some infants had similar diagnostic samples taken at their local hospital, prior to PICU admission. These results were obtained and collated for each admission where available. All patients also had routine hematology and biochemistry samples taken on admission to PICU.

### Statistical analyses

Data were expressed as descriptive statistics as number and percentage or median and interquartile range (IQR), as appropriate on Excel 2007.

Univariate analysis, Fisher exact test, chi square test and multiple logistic regression analysis were performed using SPSS 16.

## Results

We identified 274 children who fulfilled the inclusion criteria. The total number of PICU admissions in this period was 1967 patients; therefore, children with bronchiolitis constituted 13.5% of all PICU admissions.

The majority of patients (173, 63%) were males and 100 (36%) were Caucasian (Table 1). The median age of children admitted to PICU with bronchiolitis was 60 days (IQR 28–150 days). Across all years of the study, children in the first 42 days of life were the most common age group admitted. Overall, children < 1 year of age accounted for 97% of admissions with bronchiolitis.

**Table 1:** Demographics and admission characteristics of bronchiolitis-related ICU admissions, 2011–2016

Bronchiolitis-related ICU admissions (*n*)	274 (%)
Male sex	173 (63%)
Caucasian	100 (36%)
Median age in days (range)	60 (28–150 days)
Exclusively breastfed	80 (29%)
Exclusively bottle fed	174 (63%)
Median birth weight (available in only 107 patients where available)	2.3 kg
Prematurity	126 (46%)
Household smoking	32 (12%)
Previous NICU admission	85 (31%)
Pre-existing co-morbidity	103 (38%)
Family history of atopy	44 (16%)
Median (range) number of house holders	4.4 (3–13)

### Risk factors for severe disease

Children born during the UK RSV season (October to March) accounted for 173 (63%) of cases; 174 (64%) children were bottle fed; 41 (15%) children were living with a single parent; 33 (12%) of children were living in a household with at least one smoker. The mean number of householders was 4.4 with range 3–13. A positive family history of allergy was present in 44 (16%) of patients.

One hundred twenty-six (46%) of PICU admissions with bronchiolitis were ex-premature infants (≤ 37 completed weeks gestation), 30 (11%) had been born at ≤ 28 weeks gestation. Eighty-five (31%) infants had been previously admitted to a neonatal ICU; 35 (12%) infants were admitted only to a special care baby unit (SCBU). Median birth weight of all admissions was 2.1 kg (IQR 1.6–2.9 kg) in cases where birth weight data were available (*n* = 107). Median PICU admission weight was 4 kg (IQR 3–6.2 kg). Apnea was the primary cause for admission in 102 (37%) of the cases, two thirds of these being ex-premature infants (*n* = 68).

### Co-morbidities (Table 2)

Excluding extreme prematurity, previously existing co-morbidities were recorded in 103 (38%) of children, with 60% of these having more than one co-morbidity. The most frequently observed known co-morbidities were gastroesophageal reflux disease (47, 17%), cardiac anomalies (44, 16%), chronic lung disease (including bronchopulmonary dysplasia) in 36 (13%) and known neurological abnormality in 19 (7%).

**Table 2:** Co-morbidities identified in children admitted to PICU with bronchiolitis

Number	103 (38%)
Gastro-esophageal reflux disease	47 (17%)
Cardiac abnormality	44 (16%)
Chronic lung disease	36 (13%)
Neurological	19 (7%)
Airway abnormality	18 (7%)
Trisomy 21	7 (3%)
Other chromosomal abnormalities	6 (2%)
Allergy	5 (2%)
Renal anomaly	4 (1.5%)
Other syndromes	4 (1.5%)
Congenital infection	3 (1%)
Immune deficiency	3 (1%)

Please note: 60% of these patients had more than one co-morbidity

### Microbiology (Table 3)

Respiratory syncytial virus (RSV) was the most frequently isolated pathogen, being found in 165 (60%) children. In 85 children, RSV was the only organism isolated, and in 80 children, this was found in association with another pathogen. Rhinovirus was the next most frequently detected pathogen, found in 71 (26%) children, and 21 (8%) were infected with human metapneumovirus.

**Table 3:** Infectious agents identified in children with bronchiolitis

Microbiology result	Single infection	Co-infection	Total	Percent
Viruses				
RSV	85	80	165	60%
Rhinovirus	17	54	71	26%
Adenovirus	1	17	18	7%
Human Metapneumovirus	6	15	21	8%
Parainfluenza	2	7	9	3%
Influenza A	1	1	2	
Influenza B	1	0	1	
CMV	0	4	4	1%
Bacteria		95	95	36%
*H. influenzae*	0	27	27	10%
*Staph. aureus*	0	22	22	8%
Enterobacter sp.	0	14	14	5%
Streptococcus species	0	10	10	4%
*Moraxella catarrhalis*	0	10	10	4%
*Pseudomonas aeruginosa*	0	12	12	4%
Klebsella sp.	0	8	8	3%
*E. coli*	0	7	7	3%
*Candida albicans*	0	9	9	3%
Coagulase—negative Staph	0	6	6	2%
Others	0	4	4	1%

*RSV* respiratory syncytial virus, *CMV* cytomegalovirus

Detection of multiple pathogens was common and seen in 123 (45%) of children. Any virus found together with a bacterial co-isolate was recorded in 95 (36%) children. The most commonly isolated bacteria were *Haemophilus influenzae* and *Staphylococcus aureus*. The most common sites to find associated bacteria were endotracheal aspirate in 54 (19.7%) and blood culture in 19 (7%) children. No patients had bacteria as the sole organism identified as a cause of bronchiolitis. The blood culture positivity was primarily thought to be contaminants. We do not routinely carry out throat cultures in infants admitted with presumed viral bronchiolitis. No organism was detected in 28 (10%) of children.

Ten children had received palivizumab during the same season they were admitted (three had a cardiac indication; five had chronic lung disease of prematurity; two were extremely premature). Three of these had RSV infection identified as the cause of their PICU admission.

### Outcome (Table 4)

All children admitted to PICU required some form of respiratory support. The median length of PICU stay was 6.5 days (range 18 h to 52 days); 247 (90%) children required tracheal intubation and mechanical ventilation. Fifty of these were treated with high-frequency oscillatory ventilation (HFOV) for a period of their mechanical ventilation.

**Table 4:** Mode of ventilation and outcome of children admitted to paediatric intensive care with bronchiolitis

Non-invasive ventilation (CPAP) only	27 (10%)
Invasive ventilation	247 (90%)
High-frequency oscillation	50 (18%)
Median length of PICU stay (interquartile range) days	6.5 (5–10)
Need for CPAP after extubation	144 (53%)
Mortality	6 (2%)
Complications	47 (17%)

*CPAP* continuous positive airway pressure

One hundred forty-four (58%) patients who required invasive ventilation required non-invasive respiratory support with continuous positive airway pressure (CPAP) following extubation. Twenty-seven (10%) patients required only CPAP during their PICU admission.

Complications were observed in 47 (17%) infants, most of them related to their current illness or management. Twelve (4%) patients suffered with pulmonary haemorrhage within 24 h following extubation, all requiring re-intubation; 10 patients were diagnosed with co-existent shock (defined by the need for fluid resuscitation and inotropic support); 9 children had a pneumothorax at the time of admission; 4 had cardiac failure and arrthymia; and 4 had coagulopathy associated with sepsis. Ten children had vascular complications (i.e. deep venous thrombosis or arterial thrombosis).

### Predictors of length of PICU stay as a measure of severity of bronchiolitis

To identify predictors of length of PICU stay as a marker of severity of illness, we performed multiple linear regression analyses. The following factors were noted to be significantly associated with longer length of PICU stay: younger age at admission (*p* = 0.046), gestational age (*p* = 0.002); birth weight (*p* < 0.0001), invasive ventilation (*p* < 0.0001), RSV infection (*p* = 0.0065), co-morbidity (*p* = 0.0005), co-infection (*p* = 0.0001).

We also carried out further regression analysis using days of invasive ventilation days as the outcome in order to exclude bias. The high proportion of co-morbidity and prematurity in our cohort is likely to have been associated with a requirement for longer duration of invasive ventilation, requirement for non-invasive ventilatory support post-extubation and hence prolonged LOS. Following this further regression analysis, the only significant risk factor was the age at admission (*p* = 0.04).

### Differences between children with RSV and non-RSV bronchiolitis (Table 5)

To determine if infection with RSV was particularly associated with differences in outcome compared to infection with other pathogens, we compared the demographic characteristics and risk factors in children who were infected with RSV and those without RSV. RSV infection was more common in term infants than preterm; the median gestational age in children with RSV was 38 weeks, compared with 36 weeks in the non-RSV group (*p* < 0.02).

**Table 5:** Comparison between RSV positive and non-RSV children

Demographic characteristics	RSV positive (*n* = 165)	Non-RSV (*n* = 109)	*p* value
Median gestational age of admissions (weeks)	38	36	0.02
Mean weight on admission, kg	4.5 ± 2.2	5.17 ± 2.7	0.03
Median age, days	48	90	0.004
0–2 months	104	45	0.0005
3–5 months	33	28	
6–11 months	19	30	
1 2–24 months	9	6	
Male	99 (60%)	75 (70%)	0.12
Female	66 (40%)	34(30%)	
Risk factors			
Prematurity, gestational age at birth: weeks			0.03
≤ 28	11	19	
29–32	17	14	
32–35	31	13	
35–37	12	11	
Congenital heart disease	24	20	0.4
Chronic lung disease	14	22	0.0063
Other co-morbidity	90(55%)	68(62%)	0.18
Co-infection	85(52%)	53(48%)	0.6
Parameters of disease severity			
Median length of stay, (IQR [25th–75th percentile])	7 days (5–11)	6 days (4–9)	0.02
Invasive ventilatory support	152	94	0.11
Patients receiving HFOV	31	19	0.77
Patients treated only with NIV	12	15	0.08
Need for CPAP after extubation	98	48	0.01
Complications	34	15	0.15

*IQR* interquartile range, *HFOV* high-frequency oscillation ventilation, *NIV* non-invasive ventilation, *CPAP* continuous positive airway pressure

RSV was also more common in younger infants; the median age for admission was 48 days in babies with RSV, compared with 90 days in non-RSV patients (*p* < 0.004).

Seventy percent of the children with RSV bronchiolitis were ≤ 2 months of age compared with 41% of those without RSV (*p* < 0.0005). Forty-five percent of the children with RSV bronchiolitis were previously healthy, with no obvious identifiable risk factors.

Disease severity was significantly worse in children with RSV bronchiolitis in some of the parameters evaluated, including median length of PICU stay, need for continuing respiratory support after extubation and need for supplemental oxygen after extubation, and there was a trend toward more severe disease observed in other parameters, such as the need for tracheal intubation, requirement for HFOV and development of complications.

### Mortality

Six deaths in children with bronchiolitis were recorded over the study period (2.2% of all bronchiolitis-related PICU admissions). The median age of children who died was 4.5 months (range: 56–270 days). Median LOS among deaths was 20 days (range 6–63 days).

Only one child who died had no underlying co-morbidity or prematurity. This was a previously healthy child with acute adenovirus infection who died after referral for ECMO.

Four children who died were ex-premature babies; two had underlying immunodeficiency, one had trisomy 21, one had severe chronic lung disease of prematurity and one had congenital myotonic dystrophy. Two of the deaths had multiple congenital anomalies.

### Microbiology in children who died

Three (50%) children who died had RSV; one had only RSV infection; one had RSV, adenovirus and Bordetella pertussis (this patient was sent for ECMO and died on ECMO); one had RSV and *Pneumocystis jiroveci*. Three had rhinovirus; one of these had rhinovirus and *E. coli*; one had rhinovirus, adenovirus and *Enterobacter* sp.; and one had rhinovirus and *Pseudomonas aeruginosa*.

## Discussion

Our study presents a comprehensive review of the characteristics and clinical outcomes of children admitted to a single PICU in London, UK, with a clinical diagnosis of acute bronchiolitis. While only examining a single unit’s admissions, we feel this is a representative sample of admissions to PICUs in the UK. Our unit is primarily a medical PICU, taking predominantly unplanned acute admissions. The proportion of children admitted to this PICU is reflective of the data observed by Green et al., who found that infants aged < 1 year accounted for 93% of all PICU bronchiolitis admissions in England and 11.8% (95% CI 10.5 to 13.1%) of PICU admissions in England each year. They estimated the PICU admission rate for bronchiolitis to be between 1.3 and 1.6 per 1000 infants aged < 1 year [9].

In our study, infants aged < 1 year accounted for 97% of all admissions to our PICU with bronchiolitis. The majority of our cases were infants aged ≤ 2 months (*n* = 150), and 70% of these were RSV positive, suggesting that younger infants are more vulnerable to severe RSV disease, which is consistent with the findings from previous studies [12, 18, 28].The association of younger age and prematurity with an increased risk of PICU admission was not unexpected and has been well described previously.

RSV was the most common virus identified in our study (60%). In most studies, it accounts for 60–80% of bronchiolitis cases in children below 12 months of age [14, 22, 23, 26]. Rhinovirus was the second most common virus detected (26%), followed by human metapneumovirus (7%), parainfluenza, adeno- and influenza viruses (1–8%). Dual infections were found in 46% of children in our study, while it has been reported in 20–40% of patients in previous studies [14, 20, 22, 23, 26]. Some studies suggest that co-infection is associated with more severe disease [5]. However, it is sometimes hard to determine the pathogenicity of co-infecting viruses, particularly rhinovirus. Co-infection was also a significant determining factor for length of PICU stay in our study. Richard et al. [25], also reported an increased risk of admission to the ICU when a dual viral infection was found, but the population in their study included many premature infants and children with underlying chronic illnesses. In contrast, Marguet et al. [17] did not find any difference in bronchiolitis severity in patients solely infected with RSV compared to those with RSV—rhinovirus co-infection. As the numbers of children with co-infection in our cohort and in other reports are significant, efforts to determine the contribution of more than one infecting virus would be an important element in any efforts to promote antiviral or preventative therapy for bronchiolitis.

Eight percent of children in our study had rhinovirus as the only identified pathogen and 10% of children had no pathogen identified at all. This suggests that our current routine diagnostic methods are sub-optimal, and in these critical cases, it may be appropriate to carry out extended methods for pathogen detection. However, with no effective antiviral therapies yet available, the necessity to do this for diagnostic purposes, rather than for infection control or public health measures, remains unclear.

Consistent with previous studies [8, 9], the median length of PICU stay among bronchiolitis admissions was ~ 6 days. This was significantly longer in those children with RSV infection compared to the non-RSV group. As seen in these previous studies, RSV infection also appeared to be associated with relatively more severe disease, as determined by higher numbers of children requiring post-extubation respiratory support and supplemental oxygen.

Length of stay in the PICU may not be a very objective endpoint to evaluate disease severity as it is confounded by other factors such as co-morbidity. For example, patients may be observed for longer on PICU or clinicians may have a lower threshold for initiating CPAP after a period of mechanical ventilation. Therefore, duration of invasive mechanical ventilation may be a better endpoint from this point of view. Our regression analysis confirmed this, and only age on admission was found to be a statistically significant factor when allowing for pre-existing co-morbidity.

Prematurity was common in our cohort of patients, accounting for nearly half (46%) of all patients, with extremely preterm infants (≤ 28 weeks gestation) accounting for 11% of cases admitted to PICU, compared to only 3% of hospitalized children in previous studies [9, 21], although no other studies have focused purely on PICU admission with bronchiolitis. One study from Taiwan which examined risk factors associated with death in patients with severe RSV found that almost 50% of their patients admitted to their PICU were premature, and 25% had a nosocomial RSV infection [16]. None of our patients was found to have a nosocomial cause for their acute bronchiolitis illness. Moreover, we found prematurity particularly associated with admission for apnea, being found in 66% of all admissions to PICU for apnea in the context of bronchiolitis [27].

In our study, 38% of the admissions to PICU with bronchiolitis had documented co-morbidities prior to admission or discovered during their admission for bronchiolitis. This differs from previous studies which identified co-morbidity in only 24–27% of hospital admissions with bronchiolitis [8, 12] but is fewer than the study from Taiwan quoted above, where 72% of patients admitted to PICU with RSV had a co-morbidity [16]. However, this is an important message—in children admitted to PICU with bronchiolitis, a careful search for underlying cardiac, pulmonary, or neurological disease should be undertaken in what are thought to be previously healthy children. This may unmask a condition that may predispose the infant to more frequent or more severe respiratory infection.

There is evidence that children with underlying co-morbidities who should have received RSV-prophylaxis do not always receive it [1]. Our study and other studies have demonstrated that current guidelines for RSV-prophylaxis may exclude many patients who are at high risk but who do not fulfil the current guidelines for prophylaxis and suggests that further analysis to determine those patients who may benefit most from RSV-prophylaxis is required. Use of agents such as palivizumab has been shown to reduce the burden of hospitalization and mortality in bronchiolitis in a cost-effective manner [2, 19, 24]. However, if our data is representative of PICU admissions for bronchiolitis across the developed world, other factors may need to be taken into account when deciding on eligibility for palivizumab or other similar agents. Additionally, developments in RSV vaccines suggest that many admissions to PICU could be prevented by effective maternal immunization [10].

## Limitations

Our study was of children in one representative PICU and retrospectively examined data from an existing database, medical charts and diagnostic testing results. Therefore, there was some missing data and we were reliant on the accuracy of existing coding. However, we feel that our study is representative of the current situation of admissions to general PICUs with bronchiolitis in the UK.

## Conclusion

Bronchiolitis is a significant cause of PICU admission in the developed world. Most children with bronchiolitis-related PICU admission required prolonged PICU stay and invasive ventilation.

A significant proportion of children admitted to PICU with bronchiolitis had no recognized risk factors. A careful search for co-morbidity should be made in children being admitted to PICU for bronchiolitis.

Many children admitted to PICU with RSV bronchiolitis did not fall into the recognized indications for RSV-prophylaxis, suggesting that these indications should be re-evaluated.

The increasing recognition of co-infections in acute bronchiolitis may impact the design and sample size of future studies in this field.

## Abbreviations

CMV: Cytomegalovirus
CPAP: Continuous positive airway pressure
ECMO: Extracorporeal membrane oxygenation
ETT: Endotracheal tube
HFOV: High-frequency oscillatory ventilation
ICU: Intensive care unit
IQR: Interquartile range
LOS: Length of stay
NPA: Nasopharyngeal aspirate
PIC: Paediatric intensive care
PICU: Paediatric intensive care unit
RSV: Respiratory syncytial virus
